# Non-Transferrin-Bound Iron (NTBI) Uptake by T Lymphocytes: Evidence for the Selective Acquisition of Oligomeric Ferric Citrate Species

**DOI:** 10.1371/journal.pone.0079870

**Published:** 2013-11-21

**Authors:** Joao Arezes, Monica Costa, Ines Vieira, Vera Dias, Xiao L. Kong, Rui Fernandes, Matthijn Vos, Anna Carlsson, Yuri Rikers, Graça Porto, Maria Rangel, Robert C. Hider, Jorge P. Pinto

**Affiliations:** 1 Basic and Clinical Research on Iron Biology, IBMC - Instituto de Biologia Molecular e Celular, Universidade do Porto, Porto, Portugal; 2 Pharmaceutical Sciences Research Division, King's College London, London, United Kingdom; 3 ATAF, IBMC - Instituto de Biologia Molecular e Celular, Universidade do Porto, Porto, Portugal; 4 Europe NanoPort, FEI, Eindhoven, The Netherlands; 5 Clinical Hematology, CHP-HSA - Santo António General Hospital, Porto, Portugal; 6 Molecular Immunology and Pathology, ICBAS - Instituto de Ciências Biomédicas de Abel Salazar, Universidade do Porto, Porto, Portugal; 7 REQUIMTE, ICBAS - Instituto de Ciências Biomédicas de Abel Salazar, Universidade do Porto, Porto, Portugal; Istituto Superiore di Sanità, Italy

## Abstract

Iron is an essential nutrient in several biological processes such as oxygen transport, DNA replication and erythropoiesis. Plasma iron normally circulates bound to transferrin. In iron overload disorders, however, iron concentrations exceed transferrin binding capacity and iron appears complexed with low molecular weight molecules, known as non-transferrin-bound iron (NTBI). NTBI is responsible for the toxicity associated with iron-overload pathologies but the mechanisms leading to NTBI uptake are not fully understood. Here we show for the first time that T lymphocytes are able to take up and accumulate NTBI in a manner that resembles that of hepatocytes. Moreover, we show that both hepatocytes and T lymphocytes take up the oligomeric Fe3Cit3 preferentially to other iron-citrate species, suggesting the existence of a selective NTBI carrier. These results provide a tool for the identification of the still elusive ferric-citrate cellular carrier and may also open a new pathway towards the design of more efficient iron chelators for the treatment of iron overload disorders.

## Introduction

Iron, the most abundant transition metal in mammalian systems, is essential for metabolic processes, including molecular oxygen transport and DNA synthesis. Under normal conditions, iron circulates in the plasma bound to transferrin and this constitutes the major iron source for iron-avid processes, such as erythropoiesis [1]. Circulating iron which is not bound to transferrin, heme or ferritin (here designated as non-transferrin-bound iron - NTBI) becomes important in iron overload disorders, in which plasma iron is present in excess of transferrin-binding capacity [2], [3]. In contrast to transferrin-bound iron, NTBI is avidly taken up by the liver [4], [5]. The uptake of NTBI by hepatocytes is viewed as a clearance mechanism of potentially toxic circulating iron that could otherwise, due to its involvement in the formation of free oxygen radicals [2], cause damage to other cell types. However, even the robust hepatocyte has a threshold beyond which iron accumulation becomes toxic, leading to the development of liver pathologies such as fibrosis, cirrhosis and hepatocarcinoma. These conditions are hallmarks of iron overload disorders, including beta-thalassemia and hereditary hemochromatosis. Besides hepatocytes, a variety of other cell types have also been shown to take up NTBI [6]. The specific iron uptake kinetics displayed by distinct cell types [7]–[9], together with the observation of distinct patterns of affected organs in different iron overload diseases [10], all suggest that either the different cell types may differ in the expression of the same NTBI-carrier molecule(s) or that they possess different uptake systems capable of discriminating between the various circulating NTBI species. Until now little was known about the capacity of T lymphocytes to take up NTBI. As one of the major cellular components of peripheral blood, T lymphocytes can be exposed to circulating NTBI and have been for a long time proposed to act as a first physiological barrier against iron-mediated toxicity in situations of systemic iron overload (reviewed in Porto and De Sousa [11]). The first evidence suggesting a role for T lymphocytes in the modulation of NTBI deposition came from the demonstration of liver iron overload in mice deficient in CD8^+^- and total T lymphocytes [12]–[14]. In addition, a negative correlation has been consistently found between total body iron stores and CD8^+^ lymphocytes in the peripheral blood and was also documented in liver biopsies from HFE-hemochromatosis patients [15], [16], supporting their role in the protection against iron accumulation. In the present study we provide, for the first time, evidence demonstrating that T lymphocytes are able to take up and accumulate NTBI and that, like hepatocytes, selectively uptake a unique oligomeric ferric citrate species in conditions mimicking those observed during iron overload disorders, suggesting the existence of a selective NTBI carrier.

## Methods

### Ethics Statement

Animal care and procedures were in accordance with institutional guidelines. This study and all conducted experiments were approved by the IBMC.INEB Animal Ethics Committee, in accordance with the Portuguese Veterinary Director General guidelines. Peripheral Blood Mononuclear Cells and peripheral blood were obtained from patients at Santo António General Hospital (Porto, Portugal), who gave their written informed consent to participate in this study, which was approved by the Santo António Hospital Ethical Committee.

### Reagents and antibodies

^55^FeCl_3_, ^125^I-Transferrin and ^14^C-labeled citric acid were purchased from Perkin Elmer; microbead-conjugated anti-CD3, anti-CD4 and anti-CD8 antibodies were purchased from Miltenyi Biotec; sucrose and Dynasore were purchased from Sigma Chemical Co; siRNAs were purchased from Eurogentec.

### Isolation of human peripheral blood cells

Peripheral Blood Mononuclear Cells (PBMCs) were obtained from apparently healthy volunteer blood donors, randomly recruited at Santo António Hospital Blood Bank (Porto, Portugal). Cells were isolated by gradient centrifugation over Lymphoprep (Nycomed). After lysis of erythrocytes, cells were resuspended in RPMI (GibcoBRL) supplemented with 10% fetal calf serum (FCS; GibcoBRL) and plated. CD3^+^, CD4^+^ and CD8^+^ cells were purified from PBMCs using magnetic-activated cell sorting (MACS), after incubation with specific microbead- conjugated antibodies, according to manufacturer's instructions.

### Isolation of plasma from a hereditary hemochromatosis patient

Peripheral blood was collected from an iron-overloaded hereditary hemochromatosis patient at the time of a scheduled phlebotomy. Nine ml of blood were transferred to blood collection tubes with vacuette gel (Greiner) and centrifuged for 10 minutes at 3000 rpm. Plasma was collected and stored at −80°C until use. The plasma transferrin saturation was measured in Hospital Santo António, using routine procedures, and established to be of 85%.

### Isolation of mouse hepatocytes

Hepatocyte isolation was performed by collagenase perfusion, as previously described [17].

### Cell lines

The hepatoma cell line HepG2 was grown in D-MEM (GibcoBRL) containing 1% of penicillin/streptomycin/amphotericin (PSA) solution and 10% heat-inactivated fetal bovine serum (FBS).

### NTBI uptake

Uptake of non-transferrin- bound iron (NTBI) was assessed using ^55^Fe- citrate [18]. ^55^Fe- citrate stock solutions were prepared by mixing ^55^FeCl_3_ with unlabelled trisodium citrate, at different Fe∶citrate molar ratios. The pH was maintained at 7.4 and solutions were allowed to rest for 20 minutes before being diluted 33-fold in uptake medium and added to cells. Specific activity in the uptake medium was approximately 30 counts.min^−1^.pmol^−1^ Fe. Likewise, ^14^C-labelled ferric citrate was prepared by mixing ^14^C-labelled citric acid with unlabeled FeCl_3_ at different Fe∶citrate molar ratios. The entire procedure was similar to the one followed for the preparation of ^55^Fe-citrate. The specific activity in the uptake medium was approximately 25 counts.min^−1^.pmol^−1^ citrate. All Fe∶citrate solutions were prepared immediately before use and discarded after each experiment. Unless otherwise indicated, cells were depleted of intracellular transferrin by incubation for 1 h in serum-free/iron-free RPMI, washed and incubated with RPMI+20% HH plasma+5 µM ^55^Fe-citrate (as 5 µM ^55^FeCl_3_+100 µM citric acid), at 37°C. 5 µM is the typical NTBI concentration reported in sera from *thalassemia major* patients [19] and 100 µM citric acid corresponds to the levels normally present in human blood plasma [18]. The pH of the uptake medium was maintained at 7.4. After incubation, cells were washed 3× with ice-cold buffer [20 µM desferrioxamine (DFO) in PBS, pH 7.4], lysed with 0.1% NaOH, 0.1% Triton X-100 and intracellular Fe was measured in a MicroBeta Trilux β-counter (Perkin Elmer), for 1 minute. No significant changes in cell viability with iron treatments was observed, using trypan blue exclusion and maintenance of proliferative potential following activation with anti-human anti-CD3 and anti-human anti-CD28 for CD3^+^, CD4^+^ and CD8^+^ T lymphocytes (Figure S1). To distinguish between intracellular and membrane-bound Fe and citrate, cells incubated for 30 minutes with varied Fe∶citrate ratios were washed four times with PBS, pH 7.4, at 4°C, and were then incubated with 1 mg/ml of the proteolytic cocktail Pronase (Sigma) for 30 min, at 4°C. The cell suspension was centrifuged at 12,000 g for 30 s, and the supernatant (containing membrane- bound radioactivity) transferred to new tubes. Cell pellets containing ^55^Fe radioactivity were solubilized as described above, while those containing ^14^C-citrate were solubilized with 1M NaOH and neutralized with 1N HCl. The cell lysates and the supernatants were counted for ^14^C in a MicroBeta Trilux β-counter, for 5 minutes.

### Autometallography

CD3^+^-cells were sorted from PBMCs using a FITC-conjugated mouse anti-human CD3 antibody (Abcam), incubated with 5 µM Fe-citrate (5∶100) for up to 3 hours, washed and iron cellular localization analysed by autometallography coupled with transmission electron microscopy (TEM), as previously described [20]. Briefly, following each period of incubation with Fe-citrate, CD3^+^ cells were washed with washing buffer and fixed with 2% glutaraldehyde (in 0.1 M Na- cacodylate+0.1 M sucrose, pH 7.2). Cells were then submitted to sulfidation with 1% ammonium sulphide (pH 9.0) in 70% (v/v) ethanol, for 15 minutes. After three washes in water, cells were incubated in a colloid-protected developer containing gum arabic, citrate buffer (pH 3.8), hydroquinone and silver nitrate, for 25 minutes in the dark. For transmission electron microscopy, cells were washed in 50 mM sodium cacodylate (pH 7.4), incubated for 24 h in 1% OsO_4_ (prepared in 10 mM calcium chloride) and then in 1% uranyl acetate for 1 h. Following ethanol dehydration and Epon embedding, ultrathin sections were obtained and analyzed with a Jeol 1400 (60 kV) microscope equipped with a Orious 1100W CCD digital camera.

### Speciation plots for ferric citrate species

Speciation plots were developed for Fe-citrate complexes formed under different ferric ion and citrate concentrations, using the Hyperquad simulation and speciation (HySS) program [21] and iron affinity constants previously described [22], [23]. The plots report the species present at equilibrium.

### siRNA-mediated silencing

CD4^+^ and CD8^+^ cells were transiently transfected with siRNAs targeting DMT1-IRE, DMT1-non-IRE and ZIP14 mRNAs or with scrambled siRNAs (all from Eurogentec), using the Amaxa Nucleofector system (Lonza) as previously described [24]. The effect of siRNA nucleofection on target mRNA levels was quantified by qRT-PCR.

*Inhibition of endocytosis* - PBMCs and HepG2 cells were washed twice with MEM Alpha (Invitrogen) medium and pre-incubated for one hour at 37°C in. Cells were washed again with MEM Alpha medium and incubated in the presence of sucrose (0.45M) for 40 min or Dynasore (80 µM) for 30 min. Internalization of NTBI was measured as the amount of intracellular ^55^Fe levels after a 30 min incubation at 37°C with ^55^Fe-labeled NTBI (10 µM ^55^FeCl_3_, 100 uM sodium citrate) and two washes with PBS supplemented with DFO (5 µM). Cells were lysed in lysis buffer (0.1% Triton X-100, 0.1% NaOH) and the radioactive content in the lysates was measured using a beta counter, as described above. Internalization of transferrin was measured as the amount of intracellular ^125^I-transferrin after 30 min incubation with ^125^I-transferrin (2.5 µg/mL) at 37°C. After the incubation period, cells were washed once with PBS, followed by two minutes incubation at room temperature in pre-warmed citrate buffer (150 mM NaCl, 20 mM sodium citrate, pH 5.0) to strip externally bound Tf and four washes with PBS. Finally, cells were lysed in lysis buffer (0.1% Triton X-100, 0.1% NaOH) and the radioactive content in the lysates was measured with a gamma counter.

### Statistical analysis

The results are expressed as mean values +/−1 standard deviation (SD). The existence of correlations between iron uptake and concentration of Fe-citrate species was assessed by simple regression analysis, performed with STATGRAPHICS Centurion XV (Statpoint Technologies). Statistical significance was set at *P*<0.01 using Student's *t* test in Microsoft Excel (Microsoft).

## Results

### Quantification and pattern of NTBI uptake

**Figure 1:**
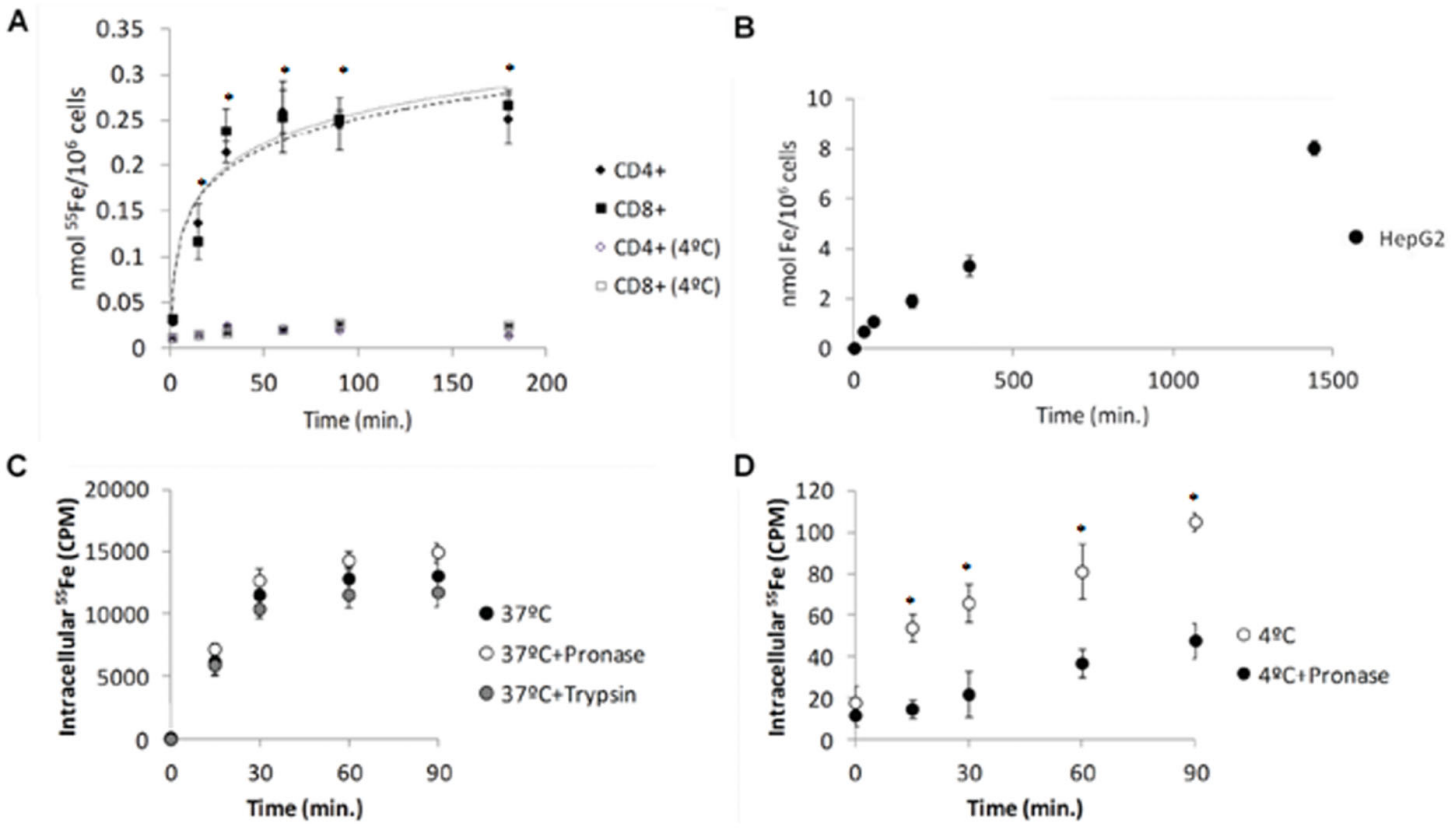
Similar patterns of NTBI uptake by T lymphocytes and hepatocytes. (A) NTBI uptake by human T-lymphocytes. CD4^+^ and CD8^+^ human T-lymphocytes were incubated with 5 µM of ^55^Fe-citrate (5∶100) at 37°C and 4°C and intracellular iron quantified at each time-point. Each point = average (n≥3) ±1SD. (B) NTBI uptake by HepG2 cells. HepG2 cells were incubated with 5 µM of ^55^Fe-citrate (5∶100) for up to 24 hours, at 37°C. Cell-associated ^55^Fe levels at each time point were measured. Each point is a mean value (n = 6) ± SD. Both T-lymphocytes and HepG2 cells are able to accumulate NTBI presenting a high rate of uptake during the first 30 minutes of incubation (C–D) Specificity of NTBI uptake. CD3^+^ cells were incubated with 5 µM of ^55^Fe-citrate (5∶100) for up to 90 min, at 37°C (C) or 4°C (D), and at each time point washed either with PBS (with or without pronase) or incubated for 15 min with serum-free RPMI with trypsin. Cell-associated ^55^Fe levels at each time point were measured. Each point is a mean value (n = 3) ± SD. The similar results obtained at 37°C together with the differences at 4°C suggest that most of the measured iron is intracellular. Statistical significance between samples at 37°C and controls at 4°C is indicated by * symbols (*p<0.01).

Both CD4^+^ and CD8^+^ human T lymphocytes accumulate approximately 250 pmol of Fe/10^6^ cells *in vitro*, when incubated with 5 µM of ^55^Fe-citrate (5∶100) at 37°C (Figure 1A). The rate of NTBI uptake is higher during the first 30 minutes of incubation (6.4 and 7.1 pmol/min./10^6^ cells, respectively for CD4^+^ - and CD8^+^ -lymphocytes), followed by a second component in which uptake is maintained at a significantly lower rate (4×10^−4^ and 7×10^−2^ pmol/min./10^6^ cells, respectively for CD4^+^- and CD8^+^- lymphocytes) until the last time point analyzed (3 hours). A plateau in intracellular iron levels is reached after approximately 60 minutes. The rate of uptake at 37°C during the initial 30 minutes was significantly higher than at 4°C (0.02 pmol/min./10^6^ cells).

To compare this pattern with a hepatocyte model, a cell type that is known to avidly acquire NTBI, we performed the same experiment in HepG2 cells. Similarly to T lymphocytes, HepG2 cells present an increased rate of NTBI uptake during the first 30 minutes (22.4 pmol/min) (Figure 1B). However, their capacity to accumulate iron is maintained until the last time point analyzed (24 hours).

Treatment with pronase and trypsin following incubation with Fe-citrate did not significantly change cell-associated radioactivity in CD3^+^ T lymphocytes (representing total T lymphocytes), suggesting that the measured Fe is mostly intracellular (Figure 1C). In contrast, at 4°C most of the NTBI is associated with the plasma membrane since cell-associated radioactivity was markedly decreased upon treatment with pronase (Figure 1D).

**Figure 2:**
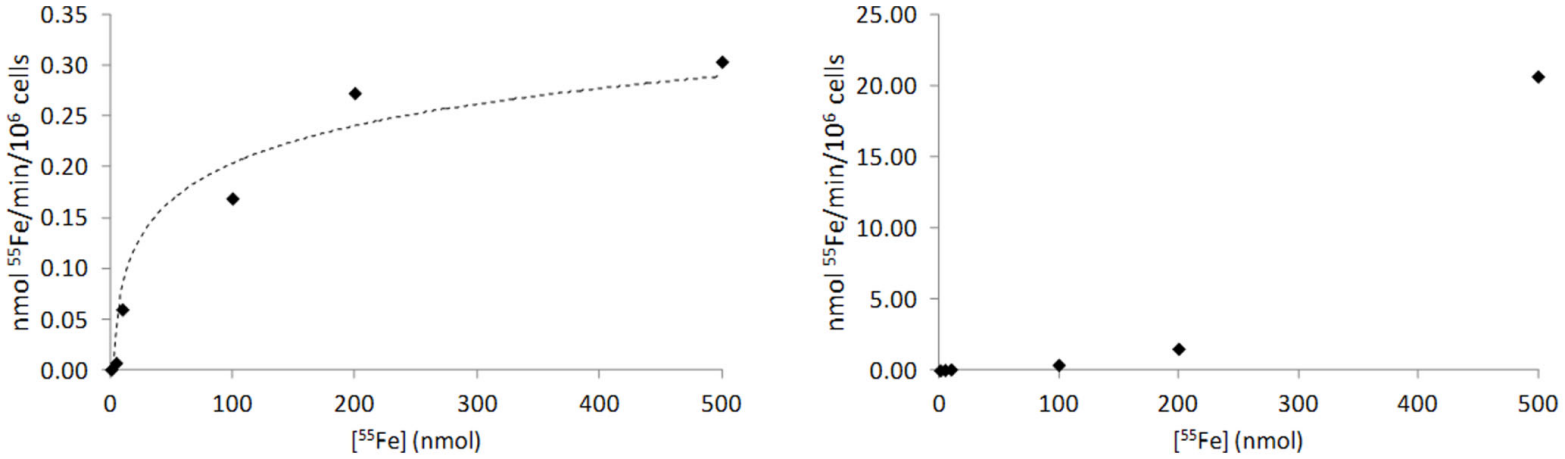
Kinetics of NTBI uptake in T lymphocytes and hepatocytes. NTBI uptake by human T lymphocytes (A) and HepG2 cells (B). Cells were incubated with different concentrations of ^55^Fe-citrate (1 µM, 5 µM, 10 µM, 100 µM, 200 µM and 500 µM) at 37°C and intracellular iron quantified at various time points (0, 15, 30, 60 and 120 min) (n = 3). The values obtained during the first 30 min of incubation, when the transport system is not saturated, were used to calculate the rate of uptake for each concentration. CD3^+^ cells reach saturation at 200 µM of Fe-citrate and present a maximum rate of 0.4 nmol/min/10^6^ cells, as opposite to HepG2 cells, which do not saturate even at 500 µM and present a faster rate of uptake (21 nmol/min/10^6^ cells).

In addition, we compared the characteristics of the Fe-citrate transport system between T lymphocytes and hepatocytes by incubating them with various concentrations of Fe-Citrate (ranging from 1 µM to 500 µM). We observed that Fe-citrate uptake in T lymphocytes reaches saturation at 200 µM, with a Michaelis constant (Km) of 92.6 nmol and maximum velocity (Vmax) of 0.4 nmol/min./10^6^ cells (Figure 2A). In HepG2 cells the uptake of Fe-citrate is faster, reaching a rate of 21 nmol/min./10^6^ cells at 500 µM (Figure 2B). The transport system was not saturated for the concentrations used, confirming that this cell type is more effective in taking up NTBI than T lymphocytes.

**Figure 3:**
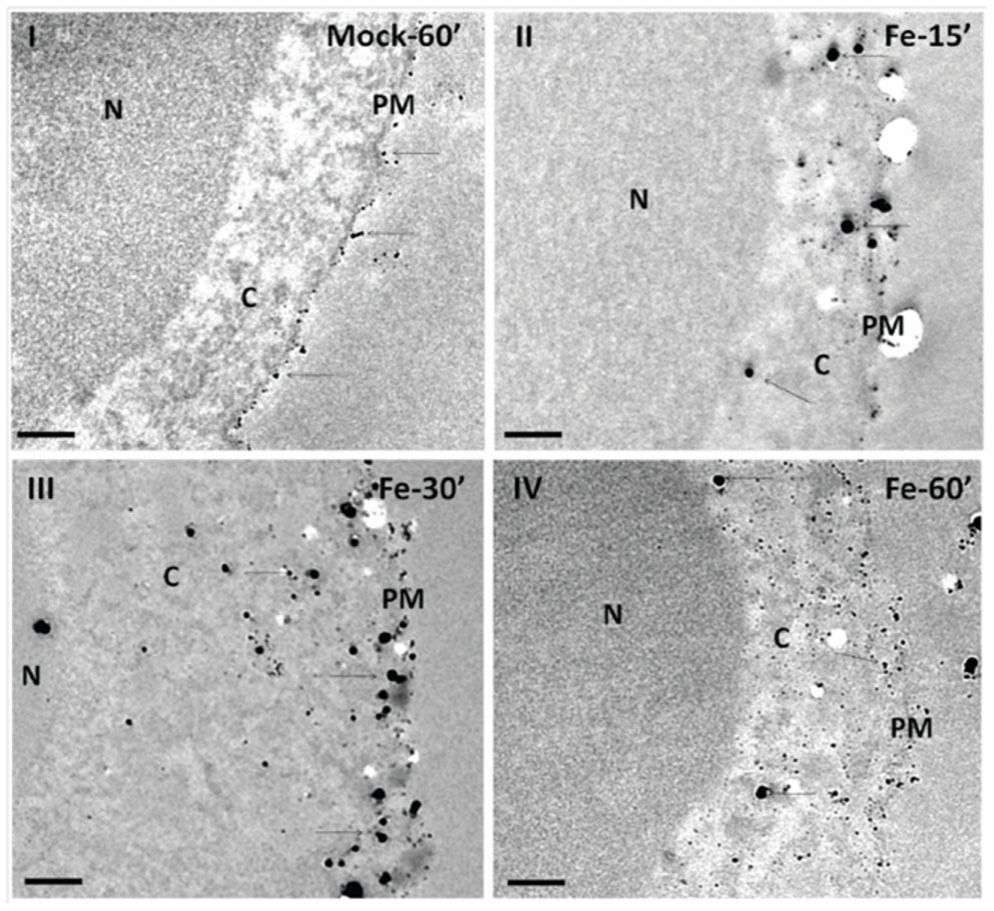
NTBI uptake by T-lymphocytes. Silver sulfide autometallography coupled with Transmission Electron Microscopy was performed in CD3^+^ T lymphocytes incubated with 5 µM of Fe-citrate (5∶100) for 15, 30 or 60 minutes. Mock control cells were incubated without Fe-citrate for 60 minutes. Arrows signal silver grains corresponding to Fe-positive particles that can be visible in the cytoplasm of cell incubated with Fe-citrate as opposite to mock control cells in which it is only associated with the plasma membrane. Highest intensity was obtained at 30 min incubation. C = cytoplasm; PM = plasma membrane; N = nucleus. Bars = 200 nm.

To further characterize NTBI uptake by T lymphocytes we used autometallography coupled with Transmission Electron Microscopy (TEM) in CD3^+^-cells exposed to 5 µM Fe-citrate (5∶100) for different time-periods. While in un-treated cells only a residual small sized silver precipitate was observed, probably corresponding to non-specific precipitation associated with the plasma membrane and never intracellularly (Figure 3-I), in cells incubated with Fe-citrate a clear granule precipitate of variable size was observed at the cell membrane and in the cytoplasm after 15 minutes of incubation (Figure 3-II). Cytoplasmic staining was even stronger at 30 minutes (Figure 3-III), while, interestingly, after 60 minutes of incubation with Fe and beyond the large-sized precipitates were almost completely replaced by smaller granules (Figure 3-IV). These granules, which resemble the Fe-positive particles observed in these cells with Energy Dispersive X-ray analysis after 24 h of incubation with Fe-citrate (Figure S2), possibly reflect the intracellular fragmentation of the larger iron-containing particles or binding to the iron-storage protein ferritin.

Altogether, these results show that T lymphocytes are able to take up and accumulate NTBI in a manner that resembles that of the HepG2 cell line.

### Ferric citrate species taken up by hepatocytes and T lymphocytes

**Figure 4:**
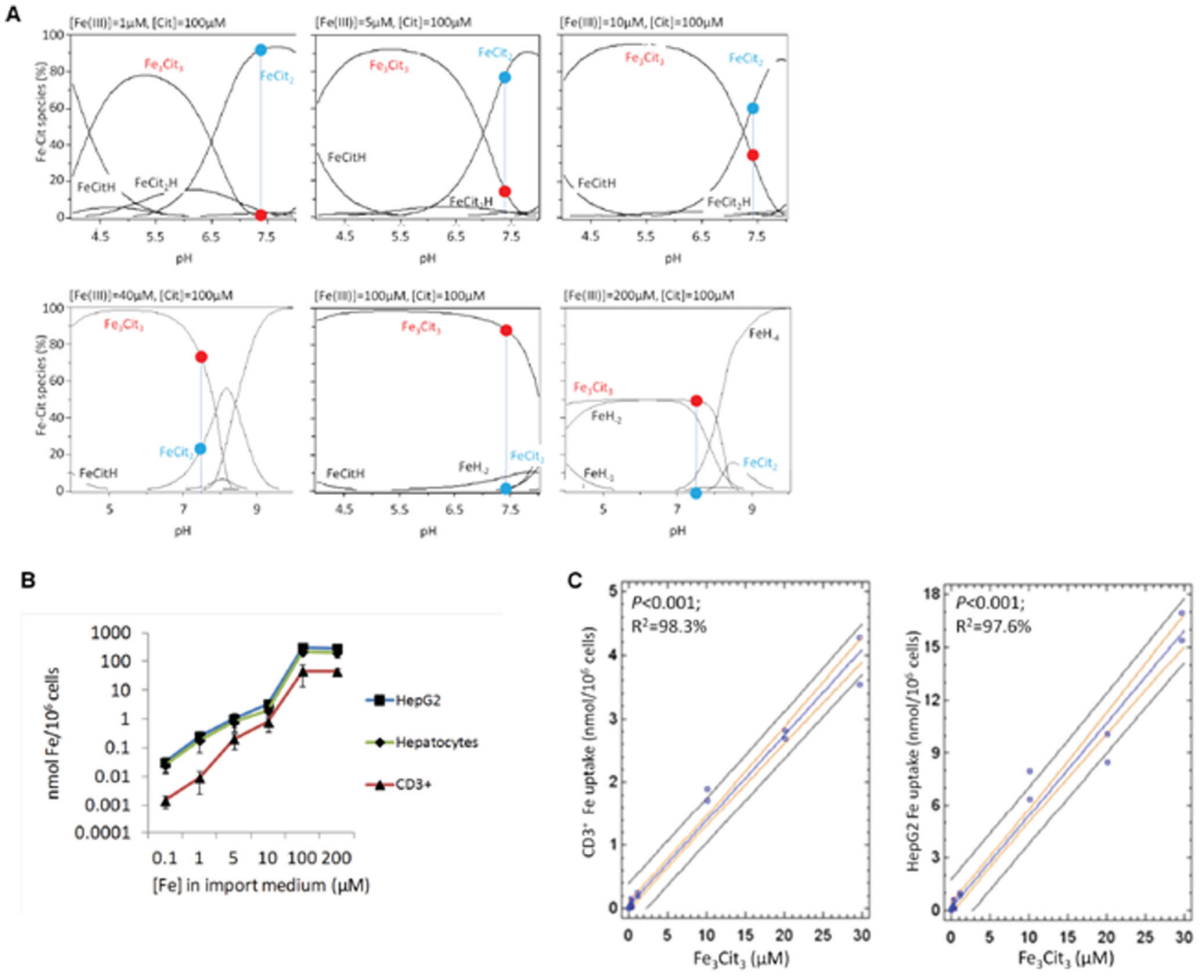
Fe uptake by T lymphocytes and hepatocytes correlates with [Fe_3_Cit_3_]. (A) Speciation plots for Fe-citrate species were calculated for Fe∶citrate ratios from 1∶100–200∶100 using the Hyperquad simulation and speciation (HySS) program. Predicted relative abundance (%) of the two most common Fe-citrate species, at pH 7.4, is marked by a blue vertical line and a red (Fe_3_Cit_3_) or blue (FeCit_2_) dot. (B) Fe uptake by T lymphocytes and hepatocytes incubated with different iron∶citrate ratios increases with the relative abundance of Fe_3_Cit_3_. Experiments were performed at least three times with three replicates per experiment. Each point represents the mean (n = 3) ±1SD. (C) Regression analysis showing a significant correlation between Fe uptake by CD3^+^ (left) and HepG2 (right) cells with predicted [Fe_3_Cit_3_] concentration at pH 7.4.

**Figure 5:**
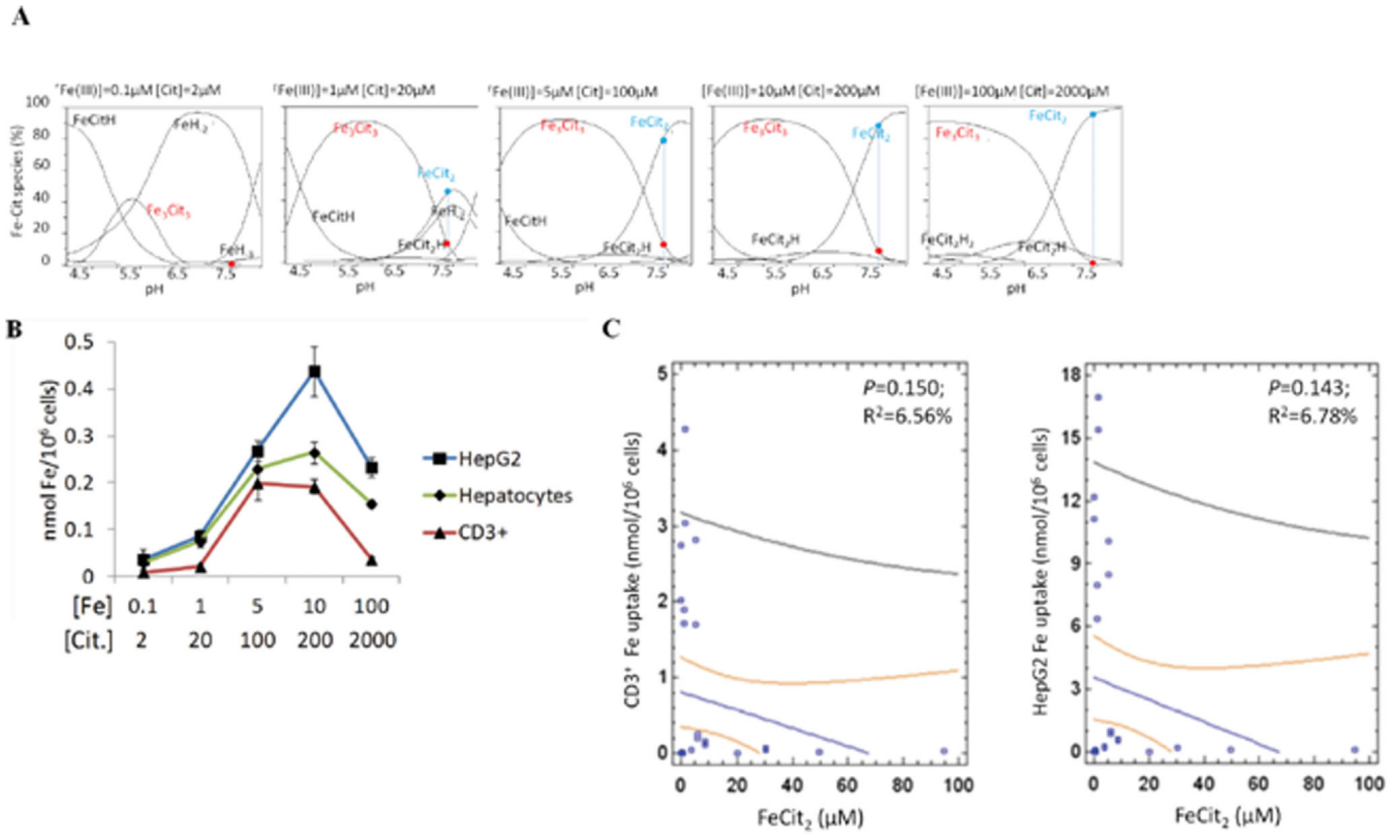
Fe uptake by T lymphocytes and hepatocytes does not correlate with [FeCit_2_]. (A) Speciation plots for Fe-citrate species, calculated for increasing Fe-citrate concentrations maintaining a constant Fe∶citrate ratio of 1∶20 using the Hyperquad simulation and speciation (HySS) program. Predicted relative abundance (%) of the two most common Fe-Cit species at pH 7.4 is marked by a blue vertical line and a red (Fe_3_Cit_3_) or blue (FeCit_2_) dot. (B) Fe uptake by CD3^+^ lymphocytes and HepG2 cells in the presence of increasing Fe-citrate concentrations, maintaining a constant Fe∶citrate ratio of 1∶20 (same conditions as in panel A). Experiments were performed at least three times with three replicates per experiment. Each point represents the mean (n = 3) ±1SD. (C) Regression analysis showing no significant correlation between Fe uptake by CD3^+^ (left) and HepG2 (right) cells with predicted [FeCit_2_] concentration at pH 7.4.

Having established that T lymphocytes and hepatocytes take up NTBI with similar patterns, we analyzed the uptake by both cell types in conditions favoring the presence of distinct Fe-citrate species. Although it is commonly accepted that Fe-citrate is one of the most relevant NTBI forms in iron overload disorders, nothing is known regarding the selectivity for particular Fe-citrate species by cells. Taking advantage of the recent development of a speciation model for Fe-citrate [22], we investigated iron uptake by hepatocytes and T lymphocytes in the presence of various Fe∶citrate ratios, for which the model predicts the formation of distinct Fe-citrate species. In the presence of 100 µM citrate, increase of Fe concentration from 0.1 to 100 µM is predicted to induce a shift from the FeCit_2_ species to the oligomeric Fe_3_Cit_3_, this latter species being essentially the only one present for iron concentrations equal or above 100 µM (Figure 4A and Table 1). When primary mouse hepatocytes, HepG2 cells and CD3^+^ lymphocytes were incubated with an equal volume of each of these Fe-citrate solutions, a dose-dependent increase in Fe uptake was observed up to 100 µM Fe for all cell types (Figure 4B), with a strong positive correlation observed between NTBI uptake and [Fe_3_Cit_3_] (Figure 4C). In contrast, when the three cell types were exposed to media predicted to contain increasing concentrations of Fe_3_Cit_3_ and FeCit_2_ (Figure 5A and Table 2), Fe uptake by hepatocytes and T cells increased only while [Fe_3_Cit_3_] was increasing and stabilized or was inhibited when [Fe_3_Cit_3_] remained stable (Figure 5A–B). The hepatoma cell line HepG2 showed a slightly different pattern of Fe uptake than hepatocytes and T lymphocytes for a particular condition in which [Fe_3_Cit_3_] remained constant and [FeCit_2_] increased almost 3-fold (10 µM Fe: 200 µM citrate). In this condition, HepG2 cells continued to increase Fe uptake, suggesting that these tumor cells may have a higher affinity for FeCit_2_ than non-transformed cells. Finally, a predicted 10-fold increase in [FeCit_2_] (with constant [Fe_3_Cit_3_]; Table 2) significantly inhibited Fe uptake by the three populations, with no correlation found between [FeCit_2_] and NTBI uptake by HepG2 and T lymphocytes (*P* = 0.15 and *P* = 0.14, respectively for CD3^+^ cells and HepG2) (Figure 5C). No association was found between the concentration of any other Fe-citrate species predicted to be present and Fe uptake (data not shown).

**Table 1:** Calculated concentrations of Fe-citrate (FeCit) species for increasing doses of Fe and 100 µM Citrate.

	0,1 µM Fe∶ 100 µM Cit	1 µM Fe∶ 100 µM Cit	5 µM Fe∶ 100 µM Cit	10 µM Fe∶ 100 µM Cit	40 µM Fe∶ 100 µM Cit	100 µM Fe∶ 100 µM Cit	200 µM Fe∶ 100 µM Cit
**Fe_3_Cit_3_ (µM)**	2.37×10^−6^	2.44×10^−3^	2.4×10^−1^	1.12	10	29.6	33
**FeCit_2_ (µM)**	9.3×10^−2^	9.22×10^−3^	3.95	6.09	8	1.65	0
**FeCit_2_H (µM)**	3.92×10^−3^	3.89×10^−2^	1.67×10^−1^	2.57×10^−1^	NA	6.97×10^−2^	NA
**FeCitH (µM)**	9.22×10^−6^	9.31×10^−5^	4.30×10^−4^	7.19×10^−4^	NA	2.14×10^−3^	NA
**FeCit_2_H_2_ (µM)**	1.22×10^−5^	1.21×10^−4^	5.19×10^−4^	7.99×10^−4^	NA	2.17×10^−4^	NA
**Fe free (µM)**	6.74×10^−12^	6.92×10^−11^	3.44×10^−10^	6.24×10^−10^	NA	2.04×10^−8^	NA
**Cit free (µM)**	4.97×10^−6^	4.88×10^−6^	4.53×10^−6^	4.18×10^−6^	NA	3.81×10^−7^	NA
**H free (µM)**	3.98×10^−2^	3.98×10^−2^	3.98×10^−2^	3.98×10^−2^	NA	3.98×10^−2^	NA

**Table 2:** Calculated concentrations of Fe-citrate (FeCit) species for increasing doses of Fe and a constant Fe∶Citrate ratio of 1∶20.

	0,1 µM Fe∶ 2 µM Cit	1 µM Fe∶ 20 µM Cit	5 µM Fe∶ 100 µM Cit	10 µM Fe∶ 200 µM Cit	100 µM Fe∶ 2000 µM Cit
**Fe_3_Cit_3_ (µM)**	6.21×10^−7^	3.91×10^−2^	2.4×10^−1^	3.15×10^−1^	4.26×10^−1^
**FeCit_2_ (µM)**	1.19×10^−3^	4.49×10^−1^	3.95	8.61	94.7
**FeCit_2_H (µM)**	5.02×10^−5^	1.90×10^−2^	1.67×10^−1^	3.63×10^−1^	3.99
**FeCitH (µM)**	5.90×10^−6^	2.35×10^−4^	4.30×10^−4^	4.71×10^−4^	5.21×10^−5^
**FeCit_2_H_2_ (µM)**	1.56×10^−7^	5.90×10^−5^	5.19×10^−4^	1.13×10^−3^	1.24×10^−2^
**Fe free (µM)**	2.16×10^−10^	9.04×10^−10^	3.44×10^−10^	1.89×10^−10^	2.11×10^−11^
**Cit free (µM)**	9.95×10^−8^	9.44×10^−7^	4.53×10^−6^	9.02×10^−6^	8.97×10^−5^
**H free (µM)**	3.98×10^−2^	3.98×10^−2^	3.98×10^−2^	3.98×10^−2^	3.98×10^−2^

**Figure 6:**
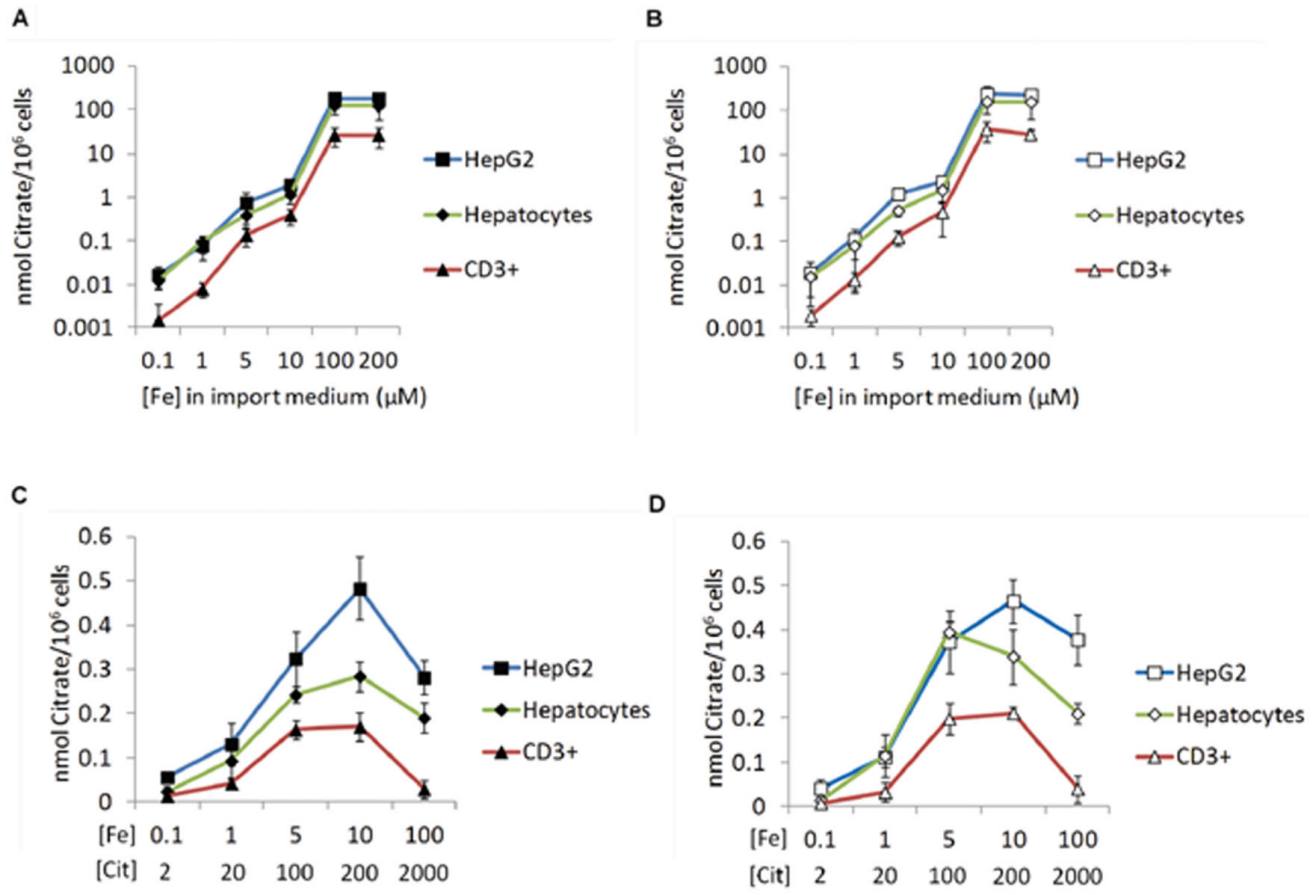
Uptake of citrate reflects the cell membrane binding capacity of each Fe-citrate species. Cells were incubated for 30∶citrate ratios and then treated with pronase. ^14^C-citrate radioactivity was measured in the cell lysates (intracellular fraction; A and C) and in the supernatants (membrane fraction; B and D). (A–B) Citrate uptake (A) and cell membrane-binding (B) by HepG2, hepatocytes and CD3^+^ lymphocytes in conditions of predicted increase of Fe_3_Cit_3_. (C–D) Citrate uptake (C) and cell membrane-binding (D) in the presence of increasing Fe-citrate concentrations, maintaining a constant Fe∶citrate ratio of 1∶20. Each point represents the mean (n = 3) ±1SD.

To test if the observed differences in the uptake of distinct Fe-citrate species could be due to differences in the binding of Fe-citrate to the cell membrane, we compared the patterns of citrate binding and internalization for FeCit_2_ and Fe_3_Cit_3_ in each cell type. Results show that the uptake (Figure 6A, C) and the binding of citrate to the cell membrane (Figure 6B, D) by the three cell types follows the same pattern observed for the uptake of ^55^Fe from Fe-citrate in each condition, suggesting that Fe_3_Cit_3_, rather than FeCit_2_, is preferentially bound to the cell membrane. Thus, differences in Fe uptake observed between each experimental condition cannot be attributed to a preferential internalization of Fe from membrane-bound Fe-citrate. It is important to state that iron precipitation, namely of ferric hydroxide, was controlled by measuring the absorbance of the ferric citrate solutions in the 280–500 nm range, with no significant decrease in the absorbance values observed for any of the solutions.

### Mechanism of NTBI uptake

**Figure 7:**
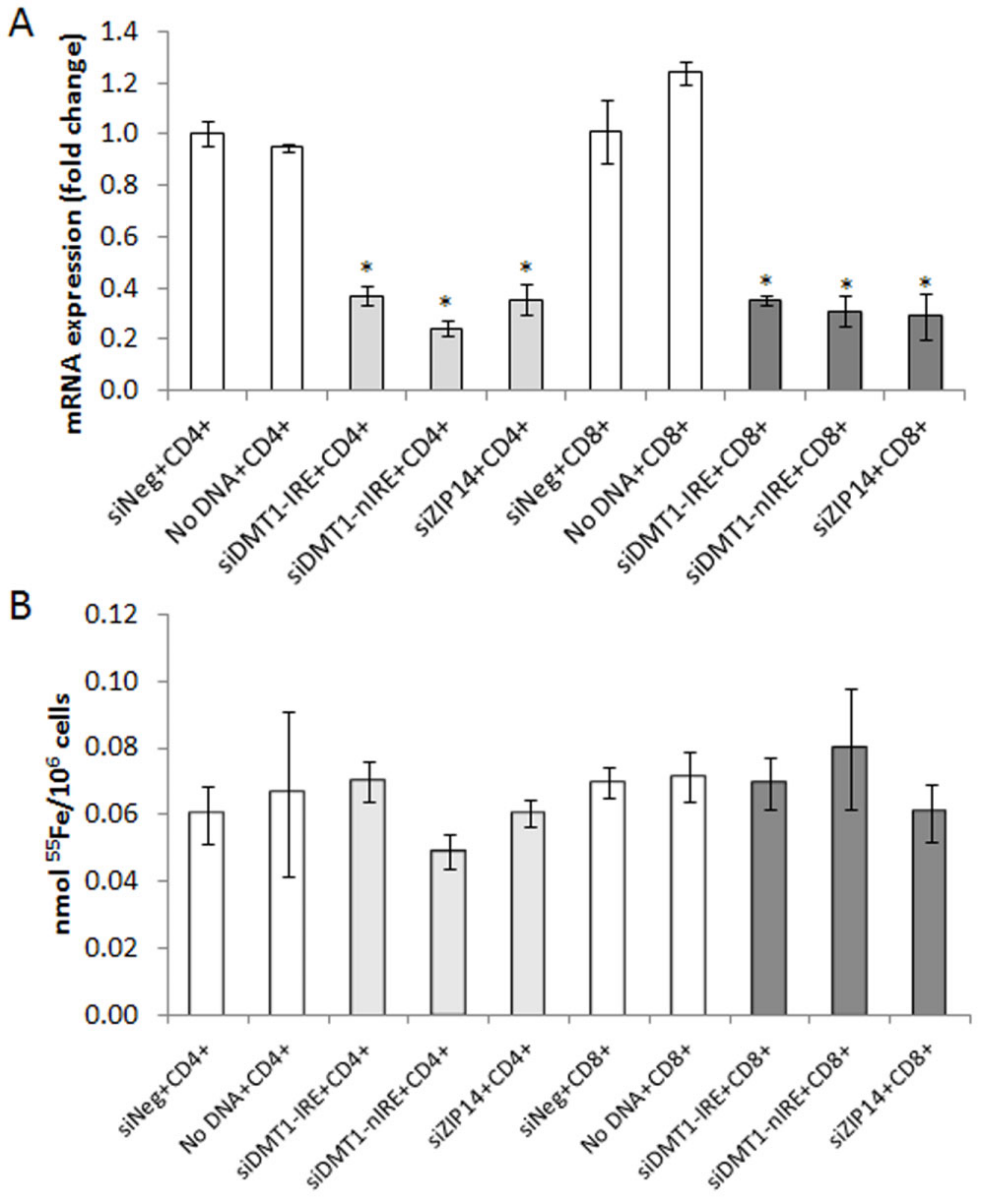
Involvement of NTBI transporters in Ferric citrate uptake by T lymphocytes. mRNA levels (A; qRT-PCR) and corresponding NTBI uptake (B) by CD4^+^ and CD8^+^ T lymphocytes following nucleofection with siRNAs specific for ZIP14, DMT1-IRE and DMT1-nIRE, with scrambled siRNAs (siNeg) or with no DNA as controls. Each column represents the mean value (n = 3) ± SD. Statistical significance between samples (grey columns) and controls (white columns) is indicated by * symbols (*p<0.01). No differences in Fe∶citrate uptake were observed after silencing DMT1 or ZIP14.

**Figure 8:**
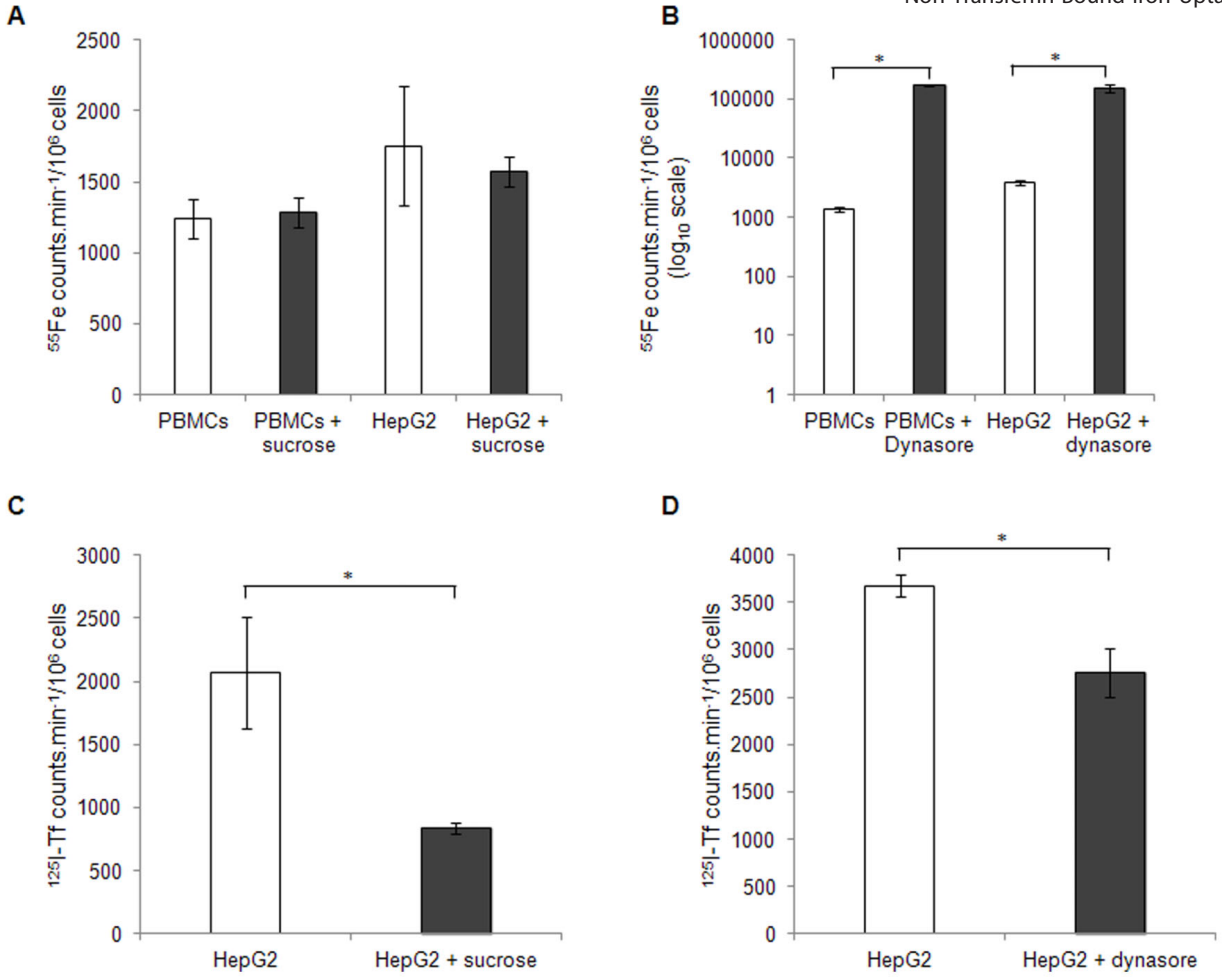
Involvement of clathrin-mediated endocytosis in NTBI uptake by PBMCs and HepG2 cells. (A–B) ^55^Fe uptake by PBMCs and HepG2 cells in the presence of 0.45M of sucrose (A) or 80 µM of Dynasore (B) to inhibit clathrin-mediated endocytosis, after incubation with 5 µM of ^55^Fe-citrate (5∶100) at 37°C (C–D) ^125^I-Transferrin internalization in the presence of 0.45M of sucrose (C) or 80 µM of Dynasore (D). Each bar represents a mean value (n = 3) ± SD. Statistical significance is indicated by * symbols (*p<0.01). The inhibition of these pathways did not prevent the uptake of NTBI in both PBMCs and HepG_2_ cells but transferrin internalization was decreased.

**Figure 9:**
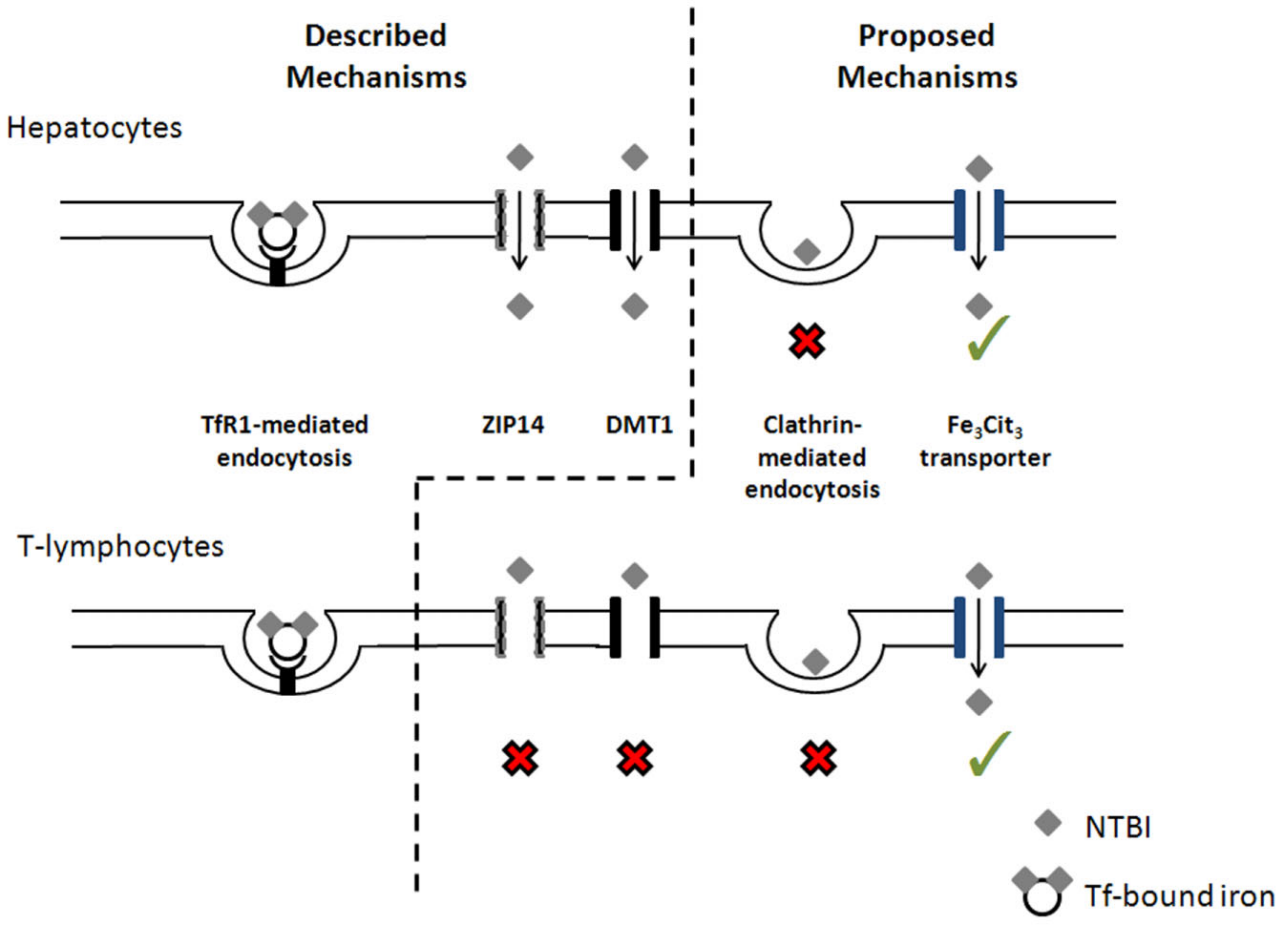
Iron uptake by T lymphocytes and hepatocytes. Transferrin-bound iron is internalized by TFR1-mediated endocytosis in hepatocytes [47] and T-lymphocytes [48]. Non-transferrin-bound iron (NTBI) is taken up by hepatocytes *via* Zrt- and Irt-like Protein 14 (ZIP14, Slc39A14; after ferric reductase-mediated reduction of Fe^3+^ to Fe^2+^) [49], [50]. Divalent metal transporter 1 (DMT1) was the first described NTBI (Fe^2+^) transporter [51], although recent data suggests it may not be important for the iron loading of the hepatocyte [52]. We hypothesize that ZIP14 and DMT1 are not involved in the uptake of ferric citrate by T lymphocytes (red×symbols). Clathrin-mediated endocytosis of ferric citrate does not occur in hepatocytes and T lymphocytes (red×symbols). T lymphocytes and hepatocytes selectively take up the oligomer Fe_3_Cit_3_, which suggests the existence of a specific transporter (green symbols).

Previous results from other groups (see Brissot *et al.* [6] for a review) have suggested that the uptake of NTBI requires specific transporters. The present evidence that NTBI uptake is essentially restricted to the Fe_3_Cit_3_ species corroborates that notion. Since DMT1 and ZIP14 are the two proteins most consistently implicated in the cellular uptake of non-heme NTBI [25], we analyzed the impact of these 2 molecules in the transport of NTBI in T lymphocytes. We found here that protein and mRNA basal expression of DMT1 and ZIP14 is either undetectable or very low in T lymphocytes and that both mRNAs are unresponsive to incubation with Fe-citrate or DFO (data not shown). Moreover, siRNA-mediated silencing of the two mRNAs to 20–40% of its basal levels had no significant effect on the NTBI uptake by CD4^+^ and CD8^+^ cells (Figure 7), ruling out a major contribution of these two proteins to NTBI uptake in these cells. To test whether endocytosis is a significant pathway for NTBI uptake by circulating blood cells, we incubated Peripheral Blood Mononuclear Cells (PBMCs) with 10 µM Fe-Citrate (1.12 µM Fe_3_Cit_3_) in the presence or absence of different endocytosis inhibitors. To assess the reach of the results, the experiments were repeated in the HepG2 cell line, a model for the hepatocyte, which accumulates the highest NTBI levels. Inhibition of the clathrin-dependent pathway using hypertonic sucrose solutions did not affect NTBI uptake by either cell type, while significantly reducing ^125^I-transferrin internalization in HepG2 cells (Figure 8A, C). Interestingly, incubation with 80 µM Dynasore, a potent inhibitor of dynamin GTPase activity [26], induced a marked increase in NTBI uptake (aprox. 128× and 40×, respectively for PBMCs and HepG2; Figure 8B), while significantly decreasing transferrin uptake (Figure 8D). Although the present results do not exclude the use of alternative endocytosis pathways for NTBI uptake by PBMCs and hepatocytes, they argue against the use of the clathrin-dependent pathway. A summary of the putative NTBI transport systems in T lymphocytes and hepatocytes and addressed in this study, as well as those previously described in the literature, is represented on Figure 9.

## Discussion

Despite the importance of NTBI in the pathophysiology of iron overload, little is still known about its chemical composition as well as about the uptake mechanisms and selectivity for distinct NTBI presentations by different cell types. Several potential iron-binding ligands are present in plasma, including citrate, acetate and albumin. Albumin, the most abundant blood plasma protein [27], has been shown to bind iron both in the presence of citrate, as a ternary complex, or in its absence [28]. Nevertheless, May et al. [29], based on the relative concentrations of potential ligands, predicted the dominant iron species in plasma to be ferric-citrate, the NTBI form used in the present study. By using a culture medium containing 20% plasma from an iron-overloaded patient, we tried to reproduce the conditions to which both hepatocytes and T lymphocytes are exposed during iron overload. Using this experimental setup we observed a strong and exclusive association between Fe uptake and the predicted presence of the Fe_3_Cit_3_ oligomer in solution, in contrast with the lack of association with FeCit_2_ or with any other ferric citrate species. Admittedly, there are differences in the media composition used for the development of the speciation models (salt-buffered aqueous solution) and for the Fe uptake assays (RPMI supplemented with 20% HH plasma). One could argue that the model predictions for a simple aqueous solution may not hold for a complex medium in which several iron binding molecules not considered in the model are present. However, significant changes in the predicted speciation between the aqueous solution and the uptake medium would only be expected if there is competition for Fe by those ligands. Under our conditions transferrin will not significantly compete with citrate, since it is 85% saturated. Similarly, acetates, pyruvates and phosphates will not compete with citrate for Fe when the citrate concentration is ≥100 µM [29], as it is the case in our experimental conditions as well as in human plasma. Finally, albumin has been described to efficiently bind iron [28] but its ability to significantly modify citrate-bound iron is dependent on its glycation and oxidation [30]. Healthy individuals have approximately only 1% of their serum albumin glycated, with this value increasing to 10% in diabetic patients [27]. No changes relatively to the 1% baseline have been described in iron-overloaded individuals, suggesting that, at least on the absence of increased glucose levels, glycated albumin will not significantly compete for iron with citrate. Albumin oxidation, on the other hand, could have a relevant effect, in face of the pro-oxidant conditions expected to arise during iron overload. Previous studies have shown that fully oxidized albumin does not show increased iron binding capacities up to 5 µM of iron, but a 2.6- fold increase was observed for 10 µM of Fe [30]. Thus, in pro-oxidant conditions albumin may show an increased capacity to modify citrate-bound concentrations, although we predict that this will only be significant for very high NTBI concentrations and extensive albumin oxidation.

The liver is one of the first and most affected organs in iron overload diseases such as beta-thalassemia and hereditary hemochromatosis. The uptake of NTBI by hepatocytes was previously described and is considered to act as a reservoir to protect other tissues from iron-mediated toxicity. The role of T lymphocytes in the uptake of NTBI has been less clear. Although the present study does not prove a role for T lymphocytes in protection from NTBI toxicity, the unequivocal demonstration of the uptake and accumulation of NTBI by T lymphocytes, together with previous results from our group showing that there is a negative correlation between the number of T lymphocytes and the severity of iron overload in HH patients [15], [16], leads us to hypothesize that these cells could act as buffers to protect other tissues from iron-mediated toxicity, confirming the postulate put forward by de Sousa and co-workers on the first demonstration of H-ferritin synthesis by human T and not B lymphocytes [31]. This early observation gains now new significance in view of recent work in conditional deletion of ferritin H in mice [32].

The mechanism for the cellular uptake of NTBI is still elusive. To address this question we analyzed the putative involvement of DMT1 and ZIP14 in the uptake of Fe-citrate by T lymphocytes. These two proteins have been previously suggested to have a role in the uptake of non-heme NTBI by hepatocytes [25]. In the case of Tlymphocytes, however, we found that the silencing of these two proteins does not have a major impact on Fe-citrate uptake, which suggests the involvement of a different, unidentified transporter that most likely favors the transport of ferric iron, differing from DMT-1 and ZIP14 which preferentially transport its ferrous form [33], [34]. A recent study has suggested that endocytic pathways mediate the uptake of NTBI by a human macrophage cell line and cardiac myoblasts/myocytes [35]. Using two different endocytosis inhibitors we demonstrated their effectiveness in inhibiting transferrin-bound-iron uptake, but not affecting NTBI internalization. The most likely explanation resides in the NTBI presentation used. The authors above used Fe-albumin as a source of NTBI and argue that this may be the main NTBI physiologically available to the cells. Our data with T lymphocytes and HepG2 cells show that, in our experimental conditions, albumin has a mild inhibitory effect on NTBI uptake (11±2%, data not shown), suggesting the inability of these cells to efficiently take up NTBI in this presentation. Although the existence of cell-specific differences has to be considered, data from previous studies showing, namely, that the rate of NTBI uptake is up to 300× faster as that from Fe-TF [36] and that, in plasma from thalassemic patients, NTBI is unlikely to be bound to albumin to a significant extent [37], support our conclusion that, at least for the cell types analyzed, endocytosis is not significantly involved in NTBI uptake. Previous studies also suggest that Fe-citrate uptake occurs by a passive, carrier-mediated process [2], [38], [39], with the identity of the carrier still unknown. The most favored hypothesis is that upon binding to its receptor, Fe-citrate is dissociated and Fe and citrate are taken up independently by cells [40], [41], with the concomitant reduction of ferric iron in the process. Our results reveal a remarkably similar pattern between Fe and citrate uptake, although a trend for a higher rate of citrate uptake over Fe was observed, supporting the hypothesis that different mechanisms control the uptake of the two components. The present results also show that the differences in Fe uptake in each experimental condition and the high correlation between Fe_3_Cit_3_ and Fe uptake are not due to a preferential uptake of membrane-bound Fe in particular conditions but instead may reflect a high specificity of a putative receptor for the oligomeric Fe-citrate species. We anticipate that this finding may be instrumental in the search for the elusive Fe-citrate transporter since it represents the identification of the ligand to which the transporter may respond, either at the level of transcription, translation or other form of modification. The high correlation between the presence of the oligomer Fe_3_Cit_3_ and Fe uptake may be surprising, as donation of Fe by smaller sized mononuclear Fe-citrate species might be expected to be more facile, particularly if the uptake involves a channel transporter. However, predictions from models and from kinetics of NTBI chelation by DFO and deferiprone suggested that the dominant species under relevant concentrations of citrate are likely to be oligomeric forms, with a molecular mass around 3.5 kDa [22], [42]. It seems thus reasonable to expect that, throughout evolution, cells have specialized in sensing and internalizing this particular species. Nevertheless, it is conceivable that particular tissues and cell types may be equipped with different cellular NTBI uptake systems, which would enable the discrimination between distinct NTBI species and could explain the different patterns of organ iron-loading observed in the various iron overload syndromes [43]. Particularly interesting would be to extend the present analysis to cardiomyocytes, a cell type in which NTBI accumulation has a particularly adverse effect [44], [45] and for which the involvement of specific NTBI transporters have been suggested [46]. Finally, the demonstration that Fe_3_Cit_3_ represents an important component of the NTBI taken up by hepatocytes and T lymphocytes could be used as a tool for optimization of chelator properties and of chelation regimens presently in use.

## Supporting Information

Figure S1 **Effect of NTBI uptake on T lymphocyte proliferation.** CD3^+^ lymphocytes were incubated with RPMI (left panel) or RPMI+ αCD3+ αCD28 (right panel), in the absence (Mock) or presence of Fe-citrate (5 or 50 µM) and cell number determined by direct counting using a Neubauer chamber every 24 hours. Figure represents the average ±1SD of two independent experiments; *P = 0.003 between Fe-citrate-treated samples and Mock (two-way ANOVA).
(TIF)

Figure S2 **Intracellular NTBI detection in T lymphocytes.** CD3^+^ cells were incubated with 5 µM of Fe-citrate (5∶100) or in Fe-Citrate-free medium (Mock) for 24 hours, followed by elemental analysis using Energy Dispersive X-ray spectroscopy. Fe-specific signals (white dots) were observed in the cytoplasm (more) and nucleus (less) of NTBI-treated (I) and not in mock cells (II), showing that this cell type is able to take up Fe-citrate. Bars = 200 nm.
(TIF)

File S1 **Supporting methods.** Experimental procedures used to analyze T lymphocyte activation and proliferation; description of the Energy Dispersive X-ray analysis technique performed for elemental mapping of iron-loaded lymphocytes.
(DOCX)
