# Supplementary figures and images for: Non-Transferrin-Bound Iron (NTBI) Uptake by T Lymphocytes: Evidence for the Selective Acquisition of Oligomeric Ferric Citrate Species
Source: PLoS One. 2013 Nov 21;8(11):e79870. doi: 10.1371/journal.pone.0079870 (PMC3836815; doi:10.1371/journal.pone.0079870)

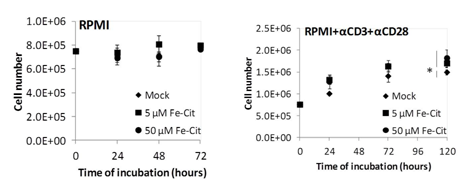

Supplement: Figure S1 — Effect of NTBI uptake on T lymphocyte proliferation. CD3+ lymphocytes were incubated with RPMI (left panel) or RPMI+ αCD3+ αCD28 (right panel), in the absence (Mock) or presence of Fe-citrate (5 or 50 µM) and cell number determined by direct counting using a Neubauer chamber every 24 hours. Figure represents the average ±1SD of two independent experiments; *P = 0.003 between Fe-citrate-treated samples and Mock (two-way ANOVA). (TIF) [file pone.0079870.s001.tif]

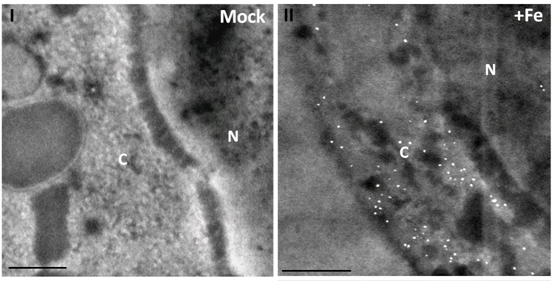

Supplement: Figure S2 — Intracellular NTBI detection in T lymphocytes. CD3+ cells were incubated with 5 µM of Fe-citrate (5∶100) or in Fe-Citrate-free medium (Mock) for 24 hours, followed by elemental analysis using Energy Dispersive X-ray spectroscopy. Fe-specific signals (white dots) were observed in the cytoplasm (more) and nucleus (less) of NTBI-treated (I) and not in mock cells (II), showing that this cell type is able to take up Fe-citrate. Bars = 200 nm. (TIF) [file pone.0079870.s002.tif]
