# Supplementary material for: Non-Transferrin-Bound Iron (NTBI) Uptake by T Lymphocytes: Evidence for the Selective Acquisition of Oligomeric Ferric Citrate Species
Source: PLoS One. 2013 Nov 21;8(11):e79870. doi: 10.1371/journal.pone.0079870 (PMC3836815; doi:10.1371/journal.pone.0079870)
Supplement: File S1 — Supporting methods. Experimental procedures used to analyze T lymphocyte activation and proliferation; description of the Energy Dispersive X-ray analysis technique performed for elemental mapping of iron-loaded lymphocytes. (DOCX) [file pone.0079870.s003.docx]

**File S1**

*T-lymphocyte activation and proliferation -* CD3^+^ lymphocytes were isolated from human PBMCs with MACS and activated using the T-Cell Activation/Expansion Kit (Miltenyi Biotec). Throughout the entire duration of the experiment, cells were maintained in serum-free RPMI with 0, 5 or 50µM Fe-Citrate (5:100 or 50:100). Serum-free conditions were used to minimize transference of Fe from citrate to transferrin. T-lymphocyte proliferation was assessed by direct counting using a Neubauer chamber at different time-points. Non-activated CD3^+^-cells were used as controls.

*Energy Dispersive X-ray analysis* - For high resolution fast elemental mapping of iron-loaded lymphocytes, a FEI Tecnai Osiris transmission electron microscope with ChemiSTEM technology was used. Images were recorded using a Fischione High Angle Annular Dark Field (HAADF) detector. A FEI super-X EDX detector was used, which provides a solid collection angle of 0.9 sterads fully integrated with the A-TWIN objective lens. The elemental maps were recorded with a 20 µs dwell time per pixel (spectra) with a 1K x 1K frame size (~20s scan time per total frame). As plastic sections are susceptible to shrinkage and beam damage, care was taken not to expose the sample outside of the acquisition period by blanking the beam when no image was acquired. Before scanning, samples were pre-eradiated in TEM mode at low magnification for 30 minutes to reach a stable state with respect to shrinkage. During and after the period of acquisition, the scanning area was assessed with respect to the contrast of the image, which was taken as a measurement of stability. No additional shrinkage or carbon deposit occurred during the scanning period as the contrast of the image did not change after each consecutive scan. We assumed therefore that, after pre-eradiation, the sample was in a stable state and no additional mass loss occurred, nor contamination deposited on the sample during acquisition, showing that both the beam current was acceptable for the imaging and the column contamination was negligible. The spectra were processed using Bruker Esprit software and the elemental map of Fe was calculated. The EDX maps were processed with an opening filter followed by a Gaussian smoothing.
